# Response to Measles, Mumps and Rubella (MMR) Vaccine in Transfusion-Dependent Patients

**DOI:** 10.3390/vaccines9060561

**Published:** 2021-05-27

**Authors:** Maddalena Casale, Nicoletta Di Maio, Valentina Verde, Saverio Scianguetta, Maria Grazia Di Girolamo, Rita Tomeo, Domenico Roberti, Saverio Misso, Silverio Perrotta

**Affiliations:** 1 Department of Women, Child and General and Specialized Surgery, University “Luigi Vanvitelli”, 80138 Naples, Italy; nicolettadimaio@virgilio.it (N.D.M.); valentina.verde@unicampania.it (V.V.); saverio.scianguetta@unicampania.it (S.S.); Domenico.Roberti@nyumc.org (D.R.); silverio.perrotta@unicampania.it (S.P.); 2 Immuno-Transfusion Service, ASL Caserta, 81031 Aversa, Italy; mg.digirolamo@gmail.com (M.G.D.G.); ritatomeo@libero.it (R.T.); saveriomisso@libero.it (S.M.)

**Keywords:** transfusion, red blood cells, measles, mumps, rubella, live attenuated vaccine

## Abstract

Measles, mumps and rubella (MMR) still determine significant morbidity and mortality, although a highly effective vaccine is available. Postponing the MMR vaccination until 6 months after the last red blood cell (RBC) transfusion is recommended, but this delay is incompatible with chronic transfusions. The present study aimed at investigating the impact of blood transfusions on the immunogenicity of the MMR vaccine. In this observational study, a group of 45 transfusion- dependent (TD) patients was compared to 24 non-transfusion-dependent (NTD) patients. Immunity to measles was achieved in 35 (78%) TD and 21 (88%) NTD subjects (p = 0.7), to mumps in 36 (80%) TD and 21 (88%) NTD subjects (p = 0.99), and to rubella in 40 (89%) TD and 23 (96%) NTD subjects (p = 0.99). No significant difference was observed in the number of non-immune individuals or those with doubtful protection between the two groups (*p* > 0.05). The mean IgG value, assayed in 50 pre-storage leukoreduced RBC units, was 0.075 ± 0.064 mg/mL, ten times lower than the level assumed in blood units and considered detrimental to the immune response in TD patients. This work shows a favorable response to MMR vaccination in TD and NTDT patients and paves the way for further larger studies assessing the impact of chronic transfusions on vaccine response.

## 1. Introduction

Measles, mumps and rubella are very contagious pathologies, and serious complications may occur even after the initial infection, such as otitis media, thrombocytopenia, immunodepression [1,2], pneumonia, meningoencephalitis [3,4], deafness, orchitis, oophoritis, mastitis, pancreatitis and fetal congenital abnormalities [5]. Morbidity and mortality rates are still extremely high, especially in low-income countries, but the burden of these infections is relevant also in industrialized regions. In 2018, more than 140,000 children died from measles, mostly under the age of five or at socio-economic or health disadvantage, and in 2019 the United States and different European countries were hit by protracted outbreaks of the disease [6,7].

The World Health Organization (WHO) has confirmed that the elimination of measles and congenital rubella is one of the top public health priorities [6]. In order to carry this forward, it is essential that, whenever possible, two doses of the MMR vaccine should be offered to all individuals to eliminate the still widespread risk of infection.

The measles, mumps and rubella (MMR) vaccine represents an extremely efficacious means of primary prevention. However, there are some conditions that require precautions in the vaccine use [8].

Administering the MMR vaccine to recipients of blood products represents a precaution because of the potential passive immunity due to the donor immunoglobulins G (IgG) content, which may compromise the vaccine efficacy. Furthermore, RBC transfusions modulate the immune system of transfusion recipients by a process defined as transfusion-related immunomodulation [9].

A 3–6 month interval is recommended between vaccination and transfusion according to the transfused blood product [10]. However, this interval is not compatible with the chronic transfusion regimes in transfusion-dependent (TD) patients.

Postponing vaccination indefinitely means that the TD subjects will not have adequate vaccine cover and this will expose them to the risk of contracting the wild-type virus, presenting serious complications, leading to an additional transfusion burden and worsening of the TD patient’s clinical condition [2,11,12].

The effect of red blood cell (RBC) transfusions on live vaccine immunization has not yet been fully determined and no specific studies have been carried out. In addition, there are significant differences in the plasma and IgG content between the various types of human blood-derived products and no data are available on leukoreduced RBC units. Specific recommendations would, therefore, help promote appropriate access to vaccination for all subjects, and in particular those fragile populations such as TD individuals.

In this study, we aimed to evaluate the serum concentrations of MMR-specific IgG antibodies in a group of TD compared to a group of non-transfusion-dependent (NTD) patients, and the IgG content in a sample of leukoreduced RBC units, in order to determine the impact of blood transfusions on the response to the attenuated live virus MMR vaccine.

## 2. Materials and Methods

In Italy, the MMR vaccine has been offered free of charge to all children since 1990. Therefore, for this study, we chose only to enroll subjects born after 1990 with official certification of having received the MMR vaccination to ensure that the MMR immunity tested in the study was due to vaccination.

This was an observational study on transfusion-dependent patients compared to non-transfusion-dependent patients, as controls, prospectively followed at the study center. All consecutive patients examined at the center between January 2018 and January 2020 were assessed for study eligibility. Eligible subjects were patients who were TD at the time at which they received the MMR vaccine, and born after 1990 with official certification of having received at least one dose of MMR vaccine. Transfusion dependency was defined as receiving ≥8 RBC transfusions per year over at least 2 years.

NTD patients who were not on a regular transfusion program, had not received any transfusion during the 6 months before MMR vaccination, and were born after 1990 with official certification of having received at least 1 dose of MMR vaccine were evaluated as controls.

Subjects were excluded for any of the following: age <1 year, receiving plasma or platelet transfusion within 7 months prior to vaccination or serology sampling, receiving non-specific intravenous Ig within 11 months preceding vaccination or serology sampling, receiving regular transfusions with washed RBCs, having a known immune deficiency, unassessed serum concentrations of measles-, mumps-, and rubella-specific IgG antibodies as per clinical practice in the study center.

For the subjects meeting inclusion criteria and enrolled into the study, we collected information concerning age, sex, hematological disease, age at first and at second dose of MMR vaccine, time elapsed between preceding and following RBC transfusion and each dose of MMR vaccine, serum concentration of MMR-specific IgG antibodies, age at which IgG-specific antibodies were tested, and time elapsed between vaccination and serology measurement of IgG-specific antibodies.

As a standard clinical practice at the center, serum concentrations of MMR-specific IgG antibodies were assayed in all patients on regular follow-up at least once and were analyzed using a commercial enzyme immune assay (EIA), and the results were classified as ‘non-immune’; ‘doubtful protection’; ‘immune’, according to the reference values for IgG antibodies (<150 mUI/mL, 150–200 mUI/mL, >200 for measles; <70 U/mL; 70–100 U/mL; >100 U/mL for mumps; and <10 UI/mL, 10–15 UI/mL, >15 UI/mL for rubella).

The levels of IgG were measured with immunoturbidimetric assay (Tina-quant^®^ Immunoglobulin G, Roche Diagnostics International Ltd, Rotkreuz, Switzerland) in supernatants from 50 pre-storage leukoreduced RBC and related whole-blood samples randomly selected in the accredited blood bank of the Immuno-transfusion Service (ASL Caserta, Italy). Pre-storage leukoreduction was obtained filtering at room temperature with a standard leukoreduction filter, Leucoflex LCRD2 (Macopharma Italia, Milan, Italy). All the leukoreduced RBC units were tested according to the national and European laws and regulations (Ministerial Decree 02/11/2015 and EDQM 20th Edition 2020). According to the recommendations for validation and quality control monitoring, the residual white blood cell (r-WBC) content was measured in the filtered RBC units using a fluorimetric method (r-WBC ADAM, Macopharma Italia, Milan, Italy). The concentrations of r-WBC were 0.08 × 106 ± 0.03 × 106.

The study was conducted in accordance with the Declaration of Helsinki and ICH guidelines for good clinical practice and approved by the institutional review board. All patients provided written informed consent.

### Statistical Analysis

In order to analyze the collected data, a descriptive statistical analysis of the variables was first carried out; quantitative variables were analyzed using central tendency indexes and variability indexes, while categorical variables were analyzed through frequencies and percentage (*n*, %).

In primary outcome variables (“immunity to measles”, “immunity to rubella”, “immunity to mumps”), the “in doubt” value was aggregated to the “non-immune” value due to the reduced number of “in doubt” cases to not compromise the reliability of the statistical tests used.

Then hypothesis tests were carried out to assess whether there were statistically significant differences between the TD and NTD groups related to both the outcome variables and the control variables.

The tests used are non-parametric due to the small number of subjects belonging to each group (TD and NTD), which would make the parametric tests substantially unreliable and therefore inapplicable.

Consequently, to evaluate whether there was a statistically significant difference in the distribution of quantitative variables in the two groups, the Mann–Whitney U test was used, while to evaluate whether there was a statistically significant association between the group of belonging and the qualitative variables a Fisher’s exact test was used. In terms of significance level, *p* < 0.05 was considered statistically significant.

The existence of a difference between gender with respect to the three immunity variables using the Fisher’s exact test and the existence of an age difference with respect to the three immunity variables using the Mann–Whitney U test was also analyzed.

Finally, a post-hoc power analysis on the three outcome variables was done to evaluate if the non-significant results could be ascribable to an insufficient sample size.

Statistical analysis of the data was performed with IBM SPSS Statistics v25 software.

## 3. Results

According to the inclusion and exclusion criteria, 45 cases and 24 controls were selected for analysis.

At diagnosis, 38 subjects (84.5%) in the TD group had transfusion-dependent thalassemia (TDT), 4 (9%) had sickle cell disease (SCD), 2 (4.5%) had congenital dyserythropoietic anemia type II (CDA II), and 1 (2%) had hereditary spherocytosis (HS). The TD patients were transfused with pre-storage leukoreduced RBCs with a mean volume (SD) of 15 mL/kg (3.34) of RBCs at an average interval of every 2–4 weeks.

In the control group, 20 subjects (83.5%) had non-transfusion-dependent thalassemia (NTDT), and 4 (16.5%) had SCD.

For the TD patients, the median time interval (IQR) between the first MMR dose and the preceding transfusion was 14 days (6–21); this was 14 days (4–20) for TD subjects receiving a single dose of the vaccine and 12 (6–35) days for those receiving both of the doses. The median time (IQR) between the vaccine dose and succeeding transfusion was 18 days (10–27); 20 days (10–29) for TD receiving a single dose of MMR; and 16 days (10–23) for the subjects receiving both of the doses.

A comparison of the number of doses of MMR, age at first dose, age at second dose, and age at serological evaluation (specific IgG analysis) is shown in Table 1. No statistically significant difference between TD and NTD subjects was seen.

The analysis of acquired specific immunity for measles in the female group resulted in 27 (79.4%) females being immune, and 7 (20.6%) were non-immune or with doubtful protection. As for the male subjects, 29 (82.9%) were immune, and 6 (17.1%) were non-immune or had doubtful protection (*p* = 0.766). Considering specific immunity for mumps, 28 (80%) in the female group were immune, and 7 (20%) were non-immune or with doubtful protection. In the male group, 29 (85.3%) were immune, and 5 (14.7%) were non-immune or had doubtful protection (*p* = 0.752). The evaluation of specific immunity against rubella showed that 31 (88.6%) female subjects were immune, and 4 (11.4%) were non-immune or had doubtful protection, while in the male group, 32 (94.1%) were immune, and 2 (5.9%) were non-immune or had doubtful protection (*p* = 0.673). As for age at the enrolment, there was no significant difference between the two groups of variables relating to immunity for measles (*p* = 0.283), mumps (*p* = 0.640) and rubella (*p* = 0.062).

**Figure 1:**
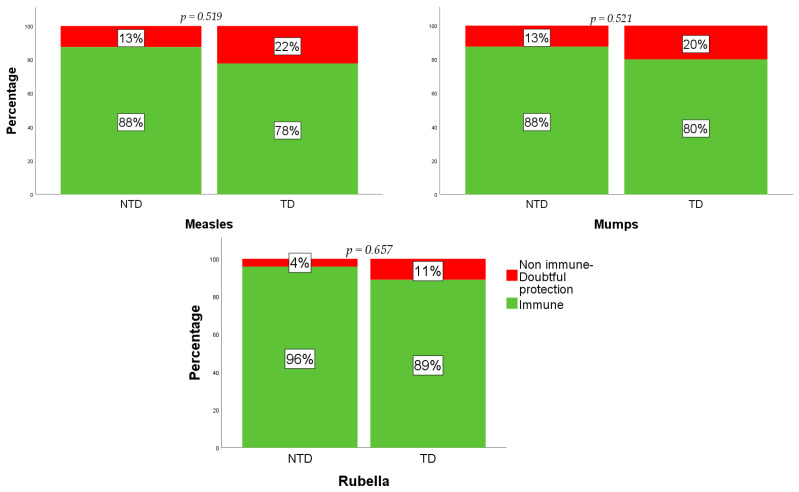
Overall response to MMR vaccine in TD patients and NTD patients.

Figure 1 summarizes the number of immune and non-immune or doubtful protection in the whole study sample. The analysis of acquired specific immunity for measles in the TD group resulted in 35 (78%) TD being immune, and 10 (22%) being non-immune or with doubtful protection. As for the NTD subjects, 21 (88%) were immune, and 3 (13%) were non-immune or had doubtful protection (*p* = 0.519). Considering specific immunity for mumps, 36 (80%) in the TD group were immune, and 9 (20%) were non-immune or with doubtful protection. In the NTD group, 21 (88%) were immune, and 3 (13%) were non-immune or had doubtful protection (*p* = 0.521). The evaluation of specific immunity against rubella showed that 40 (89%) TD subjects were immune, and 5 (11%) were non-immune or had doubtful protection, while in the NTD group, 23 (96%) were immune, 1 (4%) was non-immune, and none had doubtful protection (*p* = 0.657).

**Figure 2:**
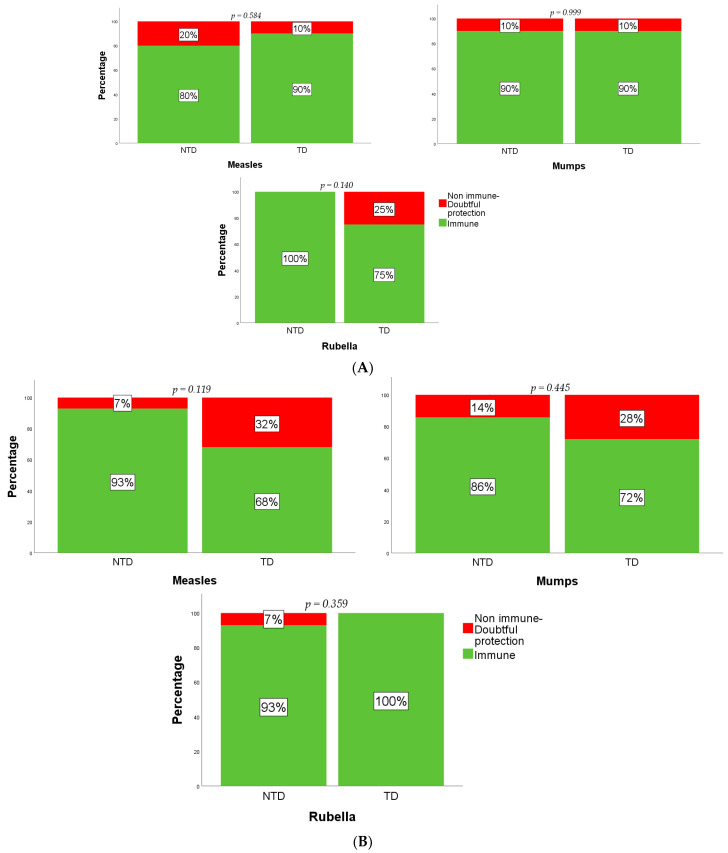
Response to MMR vaccine in TD patients and NTD patients receiving one dose (**A**) or two doses (**B**).

A comparison was then made between those subjects who received only one and those who received both doses of the MMR vaccination. No statistically significant difference, in terms of number of subjects immune, non-immune, and with doubtful protection, was observed between the TD and NTD groups (Figure 2A,B).

Among the subjects receiving both of the MMR doses, 16 (67%) TD and 11 (73%) NTD subjects achieved immunity for all three vaccine viral subunits (*p* = 0.734).

The study patients were regularly followed at the study center and no serious or relevant adverse events were recorded in the medical charts after the MMR vaccine administration.

For each whole-blood sample, the levels of IgG were within the normal range (7–16 mg/mL) and the mean value was 10.4 ± 2.1 mg/mL. In the leukoreduced RBC units, the mean IgG level was 0.75 ± 0.64 mg/mL.

A post-hoc power analysis was carried out to assess whether the non-significance of the test results could be due to an insufficient sample size of the two groups. It was decided not to carry out an a priori power analysis as the size of the two groups was not determined by the authors, instead it was a direct consequence of the study entry requirements. The post-hoc power analysis showed that the power of the test was 14.6% for the “immunity to measles” outcome, 12.8% for the “immunity to rubella” and 10.5% for the “immunity to mumps”.

## 4. Discussion

In WHO regions, every year over 25,000 children are born with TD disorders, which account for about 3.4% of the deaths in children less than five years of age [13]. TD patients have an increased risk of secondary infection due to numerous factors associated with their underlying condition (anemia, ineffective erythropoiesis, hemolysis) and therapies (iron overload, splenectomy, iron chelation therapy, central venous catheters). Furthermore, the proven immunodepressive action of viral infections, called ‘immune amnesia’, represents a particular risk of developing bacterial co-infections and serious complications related to the underlying disease [2,11,12]. Furthermore, RBC transfusions have immunological effects that result in a transfusion-related immunomodulation [9]. Until the beginning of this century, infections and their associated complications were the second most common cause of death in TD thalassemia (TDT) [14]. The fact that infections are becoming one of the main causes of death in Western countries is due, in part, to the decrease in mortality from heart disease due to iron overload [15,16]. Infections had already been reported to be the main cause of mortality among E/β thalassemia patients in Thailand twenty years ago [17], and this is also the case, more recently, in SCD patients [18]. Given this, it is essential that TD subjects have access to all appropriate preventive measures, including MMR vaccination.

American [10,19] and European [20,21,22] guidelines recommend achieving a 3–6-month free period from blood product transfusion before MMR vaccination. Given an RBC transfusion requirement of 180 mL/kg/year starting from the first years of life [23], as high as 1–4 blood units every 4 weeks, or even more frequently in the case of acute events [18,24,25], this recommended delay makes the MMR vaccination not compatible with chronic transfusions, resulting in missed preventative opportunity.

The WHO has confirmed that the elimination of measles and congenital rubella is one of their top public health priorities. Outbreaks occur where there are unvaccinated children, and recently large measles outbreaks have been reported in different countries, causing many deaths. Improving the MMR vaccination coverage and reducing the related deaths is a global imperative, and vulnerable populations should be particularly protected [6].

Studies have calculated the recommended time intervals between RBC transfusion and immunization, using an assumed level of IgG in the RBC units as high as 16 mg/mL, and the IgG half-life [10,26]. However, the quantities of IgG and plasma in each unit of human blood-derived products vary enormously so it would be useful to differentiate between the precautions to the MMR vaccine according to the product transfused.

At the moment, data are available for those subjects receiving Ig, both by the transplacental route and through high-dose therapeutic infusion for different pathologies [26,27,28,29,30,31,32,33]. A study has shown that the enzyme immunoassay antibody titers against measles, in patients who received 4 g/kg of intravenous Ig because of Kawasaki disease, was negative after 9 months following Ig infusion and the 11-month interval recommended in the United States for 2 g/kg may be longer than necessary [33].

In the absence of specific studies, the Advisory Committee on Immunization Practices (ACIP) has extended the precaution to maintain a 3–6-month transfusion-free period also in subjects receiving blood transfusions [10,19].

However, the plasma and Ig content, which could potentially interfere with the attenuated live virus vaccination, greatly varies between the various types of human blood-derived products.

The use of leukoreduced packed RBC through the pre-storage filtration of whole-blood is recommended in TD subjects [34]. The quality of leukocyte reduction is assessed by the content of residual white blood cells, but no cut off has been established for the residual amount of IgG, and a few data about the real IgG content after the pre-storage leukocyte reduction of packed RBC units are available. The mean IgG amount in leukoreduced RBC units, assayed for the first time in our study, showed a value ten times lower than that assumed, thus suggesting that the passive immunity transferred with RBC transfusion is unremarkable.

So far, only one single study has evaluated the prevalence of a protective level of specific IgG in 25 TD patients after two doses of MMR. The results showed an immunogenicity rate of 68% for measles and rubella, and 76% for mumps after a mean of 6.6 years from vaccination [35].

Any comparison between the rates of seroconversion reported in the general population is compromised by the heterogeneous methodologies and timeframes of the evaluation of protective antibodies among the different studies.

In healthy subjects, tested up to 15 years of age after two doses of the vaccine, immunogenicity was 69–98% [36,37,38,39]; lower rates were reported for mumps and higher rates for rubella. In healthy children up to 6 years of age, tested an average of 27–84 days after one or two doses of MMR vaccine, the seroconversion rate varies from 73% to 93%. More recent data have shown protection in healthy subjects in 72–95% of cases, with lower values for mumps and for those children who only received a single dose of the vaccine [40]. In young Italian adults (average age of 24.9 years), the prevalence of anti-measles IgG was >70% [41]. A study reassessing immunogenicity in the general population at 10 years after vaccination reported persistent IgG specific for the three viruses, with resistance confirmed in 86.6–96.6% of the cases [42].

In our study of TD and NTD subjects, there was no difference between the two groups in the number of vaccine doses received, the age of vaccination of the two doses, the age at which antibody testing was carried out, and the number of subjects who received only a single dose or both doses.

There was a similar long follow-up period (7.5 years) for both the TD and NTD subjects. This allows us to also demonstrate similar stability in the levels of protective IgG between TD and NTD patients, with a greater reduction in immunity against mumps than for the other two viruses, as reported in the general population.

This study showed that a chronic transfusion regimen does not significantly reduce the response to the attenuated live virus MMR vaccine administered around two weeks after transfusions and three weeks before the next. The transfusion interval was not modified for any patient awaiting vaccination. Vaccination was managed independently of the transfusion sessions.

Our data suggest that there is no reason to delay or avoid MMR vaccination in patients who receive packed RBCs, and that TD subjects should be considered a separate category of patients to those receiving other human blood-derived products.

Following the COVID-19 pandemic, the major government and medical scientific institutions in the US and in Europe have been planning how to contain infections and reduce the pressure on the national health systems. For instance, reducing the circulation of the influenza virus is a way to promote the correct diagnosis and management of suspected cases of SARS-CoV-2. This should be implemented for all preventable infectious diseases miming SARS-CoV-2 infections. Recently, it has been reported that skin lesions related to SARS-CoV-2 were misinterpreted as measles and the first case in Italy, around three months before the outbreak, remained undiagnosed for months [43]. It is, therefore, essential that, whenever possible, two doses of the MMR vaccine should be offered to all individuals to eliminate the still widespread risk of infection. Furthermore, there is a concern about the lack of efficacy of the COVID-19 vaccines because of passive immunity transferred by immunized donors, but our findings clearly show that the amount of IgGs in leukoreduced RBC units is remarkably lower than in the donors’ whole blood.

The main limitation of this study is the small patient cohort, which determines the low power of tests and the need for further studies using a larger sample. In fact, due to the low post-hoc estimated power, we cannot be sure that the non-statistically significant differences between the two groups of study is due to an equivalent response to the MMR vaccine in the two subpopulations. Furthermore, a standardized schedule for serology testing after vaccination and the dynamics of immunity development among TD patients would improve the findings of our observation. Moreover, our data are not able to verify whether the interval between vaccination and the previous and subsequent transfusions has any impact on the antibody response or on the persistence of the antibody protection within the TD study group.

The amount of donor IgG, measured for the first time in the leukoreduced RBC units, is reported to be ten times lower than the level historically assumed in blood units and considered detrimental to the immune response to vaccines in TD patients. This work shows a favorable response to MMR vaccination in TD and NTDT patients and paves the way for further larger studies assessing the impact of chronic transfusions on vaccine response.

## Figures and Tables

**Table 1:** Patients’ characteristics.

Parameters	TD (45)	NTD (24)	*p* Value
Sex (male)	24 (54%)	11 (46%)	0.62
2 doses of MMR vaccine	25 (55.6%)	14 (58.3%)	0.99
1 dose of MMR vaccine	20 (44.4%)	10 (41.7%)	0.99
Age at the enrollment in the study (years)	12 (4–22)	11 (5–14)	0.48
Age at first MMR dose (months)	18 (15–27)	16 (13–27)	0.244
Age at second MMR dose (months)	70 (65–95)	70 (65–76)	0.658
Subjects up to 6 years old receiving 1 dose of MMR vaccine	13 (62%)	7 (78%)	0.675
Overall age at serology (months)	112 (53–229)	108 (61–160)	0.537
Age at serology in subjects receiving only 1 dose of MMR vaccine (months)	84 (43–196)	128 (66–142)	0.619
Delay between MMR vaccine and serology (months)	92 (23–190)	72 (38–120)	0.77

Categorical variables are shown as frequencies and percentage (*n*, %). Continuous variables are expressed as median (IQR). TD = transfusion-dependent; NTD = non-transfusion-dependent.

## Data Availability

The data presented in this study are available on request from the corresponding author. The data are not publicly available due to ethical restrictions.

## References

[1] Lombardo D., Ciampi G., Spicuzza L. (2020). Severe and fatal measles-associated pneumonia during an outbreak in Italy: Data from the heart of the epidemic. Adv. Respir. Med..

[2] Mina M.J., Metcalf C.J., de Swart R.L., Osterhaus A.D., Grenfell B.T. (2015). Long-term measles-induced immunomodulation increases overall childhood infectious disease mortality. Science.

[3] Buchanan R., Bonthius D.J. (2012). Measles virus and associated central nervous system sequelae. Semin. Pediatr. Neurol..

[4] Bockelman C., Frawley T.C., Long B., Koyfman A. (2018). Mumps: An Emergency Medicine-Focused Update. J. Emerg. Med..

[5] Bouthry E., Picone O., Hamdi G., Grangeot-Keros L., Ayoubi J.-M., Vauloup-Fellous C. (2014). Rubella and pregnancy: Diagnosis, management and outcomes. Prenat. Diagn..

[6] Orenstein W.A., Cairns L., Hinman A., Nkowane B., Olivé J.-M., Reingold A.L. (2018). Measles and Rubella Global Strategic Plan 2012–2020 midterm review report: Background and summary. Vaccine.

[7] WHO Website. https://www.who.int/news/item/05–12–2019-more-than-140–000-die-from-measles-as-cases-surge-worldwide.

[8] Tischer A., Gerike E. (2000). Immune response after primary and re-vaccination with different combined vaccines against measles, mumps, rubella. Vaccine.

[9] Youssef L.A., Spitalnik S.L. (2017). Transfusion-related immunomodulation: A reappraisal. Curr. Opin. Hematol..

[10] Kroger A.T., Atkinson W.L., Marcuse E.K., Pickering L.K. (2006). General recommendations on immunization: Recommendations of the Advisory Committee on Immunization Practices (ACIP). MMWR. Recomm. Rep..

[11] Casale M., Perrotta S. (2011). Splenectomy for hereditary spherocytosis: Complete, partial or not at all?. Expert Rev. Hematol..

[12] Franchini M., Forni G.L., Marano G., Cruciani M., Mengoli C., Pinto V., de Franceschi L., Venturelli D., Casale M., Amerini M. (2019). Red blood cell alloimmunisation in transfu-sion-dependent thalassaemia: A systematic review. Blood Transfus..

[13] Modell B., Darlison M. (2008). Global epidemiology of haemoglobin disorders and derived service indicators. Bull. World Health Organ..

[14] Borgna-Pignatti C., Rugolotto S., De Stefano P., Zhao H., Cappellini M.D., Del Vecchio G.C., Romeo M.A., Forni G.L., Gamberini M.R., Ghilardi R. (2004). Survival and complications in patients with thalassemia major treated with transfusion and deferoxamine. Haematologica.

[15] Casale M., Filosa A., Ragozzino A., Amendola G., Roberti D., Tartaglione I., De Michele E., Cozzolino D., Rispoli G., Palmieri F. (2018). Long-term improvement in cardiac magnetic resonance in β-thalassemia major patients treated with deferasirox extends to patients with abnormal baseline cardiac function. Am. J. Hematol..

[16] Mancusi S., La Manna A., Bellini G., Scianguetta S., Roberti D., Casale M., Rossi F., Della Ragione F., Perrotta S. (2013). HNF-1β mutation affects PKD2 and SOCS3 expression causing renal cysts and diabetes in MODY5 kindred. J. Nephrol..

[17] Wanachiwanawin W. (2000). Infections in E-beta thalassemia. J. Pediatr. Hematol. Oncol..

[18] Russo G., De Franceschi L., Colombatti R., Rigano P., Perrotta S., Voi V., Palazzi G., Fidone C., Quota A., Graziadei G. (2019). Current challenges in the management of patients with sickle cell disease—A report of the Italian experience. Orphanet J. Rare Dis..

[19] McLean H.Q., Fiebelkorn A.P., Temte J.L., Wallace G.S. (2013). Prevention of measles, rubella, congenital rubella syndrome, and mumps, 2013: Summary recommendations of the Advisory Committee on Immunization Practices (ACIP). MMWR. Recomm. Rep..

[20] Ministero della Sanità, Circolare n° 12 del 13 luglio 1999, “Controllo ed Eliminazione di Morbillo, Parotite e Rosolia Attraverso la Vaccinazione”. http://www.salute.gov.it/imgs/C_17_normativa_86_allegato.pdf.

[21] Arvas A. (2014). Vaccination in patients with immunosuppression. Türk Pediatri Arşivi.

[22] Public Health England Greenbook–Immunisation against Infectious Disease. 17 December 2013. https://www.gov.uk/government/publications/immunisation-against-infectious-disease-the-green-book-front-cover-and-contents-page.

[23] Casale M., Marsella M., Ammirabile M., Spasiano A., Costantini S., Cinque P., Ricchi P., Filosa A. (2019). Predicting factors for liver iron overload at the first magnetic resonance in children with thalassaemia major. Blood Transfus..

[24] De Franceschi L., Lux C., Piel F.B., Gianesin B., Bonetti F., Casale M., Graziadei G., Lisi R., Pinto V., Putti M.C. (2019). Access to emergency departments for acute events and identification of sickle cell disease in refugees. Blood.

[25] Gianesin B., Pinto V.M., Casale M., Corti P., Fidone C., Quintino S., Voi V., Forni G.L. (2020). Manual erythroexchange in sickle cell disease: Multicenter validation of a protocol predictive of volume to exchange and hemoglobin values. Ann. Hematol..

[26] Siber G.R., Werner B.G., Halsey N.A., Reid R., Almeido-Hill J., Garrett S.C., Thompson C., Santosham M. (1993). Interference of immune globulin with measles and rubella immunization. J. Pediatr..

[27] Halsey N.A., Boulos R., Mode F., Andre J., Bowman L., Yaeger R.G., Toureau S., Rohde J., Boulos C. (1985). Response to Measles Vaccine in Haitian Infants 6 to 12 Months Old. N. Engl. J. Med..

[28] Albrecht P., Ennis F.A., Saltzman E.J., Krugman S. (1977). Persistence of maternal antibody in infants beyond 12 months: Mechanism of measles vaccine failure. J. Pediatr..

[29] Tacke C.E., Smits G.P., van der Klis F.R., Kuipers I.M., Zaaijer H.L., Kuijpers T.W. (2013). Reduced serologic response to mumps, measles, and rubella vaccination in patients treated with intravenous immunoglobulin for Kawasaki disease. J. Allergy Clin. Immunol..

[30] Velasquez D.E., Parashar U., Jiang B. (2017). Decreased performance of live attenuated, oral rotavirus vaccines in low-income settings: Causes and contributing factors. Expert Rev. Vaccines.

[31] Kaplan J.E., Nelson D.B., Schonberger L.B., Hatch M.H., Monath T.P., Lazuick J.S., Calisher C.H., Rosa F.W. (1984). The effect of immune globulin on the response to trivalent oral poliovirus and yellow fever vaccinations. Bull. World Heal. Organ..

[32] Ruderman J.W., Barka N., Peter J.B., Stiehm E.R. (1991). Antibody response to MMR vaccination in children who received IVIG as neonates. Am. J. Dis. Child..

[33] Miura M., Katada Y., Ishihara J. (2004). Time interval of measles vaccination in patients with Kawasaki disease treated with additional intravenous immune globulin. Eur. J. Nucl. Med. Mol. Imaging.

[34] Cappellini M.D., Cohen A., Porter J., Taher A., Viprakasit V., Cappellini M.D., Cohen A., Porter J., Taher A., Viprakasit V. (2014). Guidelines for the Management of Transfusion Dependent Thalassaemia (TDT).

[35] Zabeida A., Lebel M.H., Renaud C., Cloutier M., Robitaille N. (2019). Reevaluating immunization delays after red blood cell transfusion. Transfusion.

[36] Ma S.-J., Li X., Xiong Y.-Q., Yao A.-L., Chen Q. (2015). Combination Measles-Mumps-Rubella-Varicella Vaccine in Healthy Children. Medicine.

[37] Davidkin I., Jokinen S., Broman M., Leinikki P., Peltola H. (2008). Persistence of measles, mumps, and rubella antibodies in an MMR-vaccinated cohort: A 20-year follow-up. J. Infect. Dis..

[38] Demicheli V., Rivetti A., Debalini M.G., Di Pietrantonj C. (2012). Vaccines for measles, mumps and rubella in children. Cochrane Database Syst. Rev..

[39] Di Pietrantonj C., Rivetti A., Marchione P., Debalini M.G., Demicheli V. (2020). Vaccines for measles, mumps, rubella, and varicella in children. Cochrane Database Syst. Rev..

[40] Bianchi F.P., De Nitto S., Stefanizzi P., LaRocca A.M.V., Germinario C.A., Tafuri S. (2019). Immunity to rubella: An Italian retrospective cohort study. BMC Public Health.

[41] Anichini G., Gandolfo C., Fabrizi S., Miceli G.B., Terrosi C., Savellini G.G., Prathyumnan S., Orsi D., Battista G., Cusi M.G. (2020). Seroprevalence to Measles Virus after Vaccination or Natural Infection in an Adult Population, in Italy. Vaccines.

[42] Carryn S., Feyssaguet M., Povey M., Di Paolo E. (2019). Long-term immunogenicity of measles, mumps and rubella-containing vaccines in healthy young children: A 10-year follow-up. Vaccine.

[43] Amendola A., Bianchi S., Gori M., Colzani D., Canuti M., Borghi E., Raviglione M.C., Zuccotti G., Tanzi E. (2021). Evidence of SARS-CoV-2 RNA in an Oropharyngeal Swab Specimen, Milan, Italy, Early December 2019. Emerg. Infect. Dis..

